# Drug Action

**Published:** 1899-12

**Authors:** S. G. Hollingsworth

**Affiliations:** Brazil, Ind.


### DRUG ACTION.

S. G. Hollingsworth, M. D., Brazil, Ind.

It is known, though not generally made use of or even recognized, that every active drug has at least two different effects upon the human body, and that these effects may be, and are directly opposite or antagonistic if the drug is taken in the minimum and then in the maximum dose. For example—Opium introduced into the system in very small quantities causes excitation of the nervous system, while in large doses it is a powerful sedative; Strychnine in minute doses is an antispasmodic, while large doses cause tetanic convulsions. It is given to cure constipation in the former, and will produce constipation in the latter dose. Study any remedy and satisfy yourself of this dual nature of drugs.

In the homœopathic materia medica how often we find symptoms like the following: "Pulse, quick, strong, slow, feeble." One prover has used a large, the other a small dose; or, what is the same, a large dose was taken and the primary and secondary actions of the drug, which are opposite, were recorded. A practical materia medica should indicate whether the symptoms were caused by a large or by a small quantity of the drug; for, if we admit that a drug has two actions which are diametrically opposed to each other, it is very certain that a small dose prescribed according to the law *Similia Similibus Curantur* will not have the same effect on the patient who presents symptoms similar to those caused by the small dose, and on the patient who presents symptoms similar to those caused by the large dose of the same drug. This would mean that the small dose prescribed would have two directly opposite actions, and it has never yet been shown that a drug given in a specified quantity at different times would produce opposite effects.

Probably the majority of symptoms recorded in our materia medica were obtained by proving with a large dose. Then, it is evident from the foregoing that the symptoms obtained are opposite to those caused by small dose. Now, a patient presents the symptoms similar to those caused by the drug in large doses, and, according to our law, we give him the same remedy, but in a *small dose*, which is in reality *antagonistic* to the symptoms presented. A great many theories have been promulgated in an effort to explain the action of the indicated remedy. For instance, Hahnemann in *The Organon*, says, that a drug disease similar to the actual disease is set up and that while the vital force expends itself on the artificial disease, nature overcomes the actual disease. Others have advocated the "Vibration Theory," etc., but the above simple explanation seems to me the most tenable and satisfactory and furthermore can be proved.

To every unprejudiced physician it is obvious that cures are made by men of all other schools, even though the remedy is applied empirically. As there can be but one combination of elements in the production of water, so can there be but one law of cure; but there are different methods used in combining those elements. Our friends, the "Regulars," have found by experience that Strychnine in small doses will cure constipation where there is a diminution of the peristaltic action of the intestines, thereby unconsciously using the indicated homœopathic remedy. In most cases, however, a drug *antagonistic* to the symptoms presented is prescribed in *large doses* and often a cure is the result. But why not, by following the homœopathic law, place the practice of medicine on a scientific basis, do away with dangerous drugging, reduce expensive drug bills, be able to prescribe accurately for a disease in its incipiency, and still cure the patient, if he is curable, in the shortest and most gentle manner possible.—*The Medical Visitor.*

